# Complications and local recurrence of chondrosarcoma and chordoma treated by total tumor resection in thoracic and lumbar spine

**DOI:** 10.1186/s12891-024-07353-w

**Published:** 2024-03-26

**Authors:** Jiacheng Liu, Panpan Hu, Zhongjun Liu, Feng Wei

**Affiliations:** Department of Orthopedics and Beijing Key Laboratory of Spinal Disease Research, Peking University Third Hospital, 49 North Garden Rd, Haidian District, Beijing, 100191 China

**Keywords:** Spinal tumor, Chondrosarcoma, Chordoma, En bloc resection, Complication, Local recurrence

## Abstract

**Background:**

En bloc resection of spinal tumors is challenging and associated with a high incidence of complications; however, it offers the potential to reduce the risk of recurrence when a wide margin is achieved. This research aims to investigate the safety and efficacy of en bloc resection in treating thoracic and lumbar chondrosarcoma/chordoma.

**Methods:**

Data from patients diagnosed with chondrosarcoma and chordoma in the thoracic or lumbar region, who underwent total en bloc or piecemeal resection at our institution over a 7-year period, were collected and regularly followed up. The study analyzed overall perioperative complications and compared differences in complications and local tumor recurrence between the two surgical methods.

**Results:**

Seventeen patients were included, comprising 12 with chondrosarcoma and 5 with chordoma. Among them, 5 cases underwent intralesional piecemeal resection, while the remaining 12 underwent planned en bloc resection. The average surgical time was 684 min (sd = 287), and the mean estimated blood loss was 2300 ml (sd = 1599). Thirty-five complications were recorded, with an average of 2.06 perioperative complications per patient. 82% of patients (14/17) experienced at least one perioperative complication, and major complications occurred in 64.7% (11/17). Five patients had local recurrence during the follow-up, with a mean recurrence time of 16.2 months (sd = 7.2) and a median recurrence time of 20 months (IQR = 12.5). Hospital stays, operation time, blood loss, and complication rates did not significantly differ between the two surgical methods. The local recurrence rate after en bloc resection was lower than piecemeal resection, although not statistically significant (*P* = 0.067).

**Conclusions:**

The complication rates between the two surgical procedures were similar. Considering safety and local tumor control, en bloc resection is recommended as the primary choice for patients with chondrosarcoma/chordoma in the thoracic and lumbar regions who are eligible for this treatment.

## Background

Chondrosarcoma is a malignant tumor derived from the cartilaginous matrix, and it can be subclassified as either a primary malignant bone tumor or a secondary malignant transformation of an underlying enchondroma or osteochondroma. This tumor comprises 20–27% of malignant bone tumors [1, 2]. Chordoma is a rare primary malignant tumor presumed to originate from the residual embryonic notochord, accounting for 1–4% of skeletal malignancies [3, 4]. Chondrosarcoma typically manifests in long bones or the pelvis, with 6.5–10% of cases arising in the mobile spine [5]. In contrast, chordoma exclusively occurs in axial bones, with 41.1% in the skull base, 31.5% in the sacrum, and 27.4% in the mobile spine [6]. Patients with spinal chondrosarcoma or chordoma commonly present with local pain and/or neurological deficits. Neurological dysfunction can vary from radicular pain to paralysis, depending on the extent of tumor mass compression on nerve roots or the spinal cord [5, 7, 8].

Traditional radiation therapies, such as photon radiation, and chemotherapy are generally considered ineffective for treating chondrosarcoma and chordoma in the spine. Surgical resection remains the preferred treatment option [9]. En bloc resection with a wide margin is the favored surgical procedure, as prior studies have demonstrated an increased risk of locoregional recurrence with intralesional tumor excision [4, 7, 8, 10–12]. Achieving en-bloc resection of spinal tumors is technically challenging due to the complexity of spinal anatomy and proximity to vital organs such as vessels and the spinal cord. Previous reports indicate a complication rate ranging from 46.2 to 86.7% for en bloc spinal tumor resection [13–18]. In a previous investigation focusing on surgical safety for spinal giant cell tumors, 30 out of 41 patients underwent en bloc resection, experiencing increased intraoperative blood loss and perioperative complications [19].

Given the shared attributes of spinal chondrosarcoma and chordoma, including tumor location, clinical presentation, histopathological and radiological findings, as well as tumor growth and invasion patterns [20], we have grouped these two neoplasms into the same cohort for the present study. Our aim is to explore surgical safety and local tumor control outcomes in this study.

## Methods

### Patients’ inclusion

Patients diagnosed with chondrosarcoma and chordoma in the thoracic and lumbar spine who underwent total resection surgery at our institution between January 2013 and December 2020 were included in this study. Inclusion criteria comprised: (1) spinal lesions treated at our spinal center; (2) tumor diagnosis of chondrosarcoma or chordoma confirmed by pre- or post-operative pathological examination; (3) follow-up at three, six, and 12-months post-surgery, followed by annual assessments; (4) complete access to all clinical data. Exclusion criteria included: (1) lack of postoperative follow-up; (2) palliative surgery instead of resection surgery; (3) absence of a definitive pathological diagnosis. Out of 95 patients diagnosed with primary thoracic and lumbar tumors and undergoing total tumor resection during the specified period, 17 patients met the inclusion/exclusion criteria. Approval and supervision for the retrospective study were obtained from our institutional ethics committee board, and informed consent was secured from all participants.

### Surgical procedures

Preoperative imaging were employed to determine the Weinstein-Borinani-Biagini (WBB) stage of the patients [21]. Resection surgery was carried out through en-bloc or piecemeal approaches, with en-bloc resection being the predominant method in most cases, following Boriani et al’s recommendations [21–23]. Spinal reconstruction utilized titanium-alloy 3D-printing prostheses or titanium mesh, with pedicle screws and titanium rods used for posterior fixation. All procedures were performed by the same surgical team. Follow-up timelines were established at three, six, and 12-months post-surgery, followed by annual assessments. Patient information, surgical data, and follow-up events were meticulously recorded and analyzed.

**Fig. 1:**
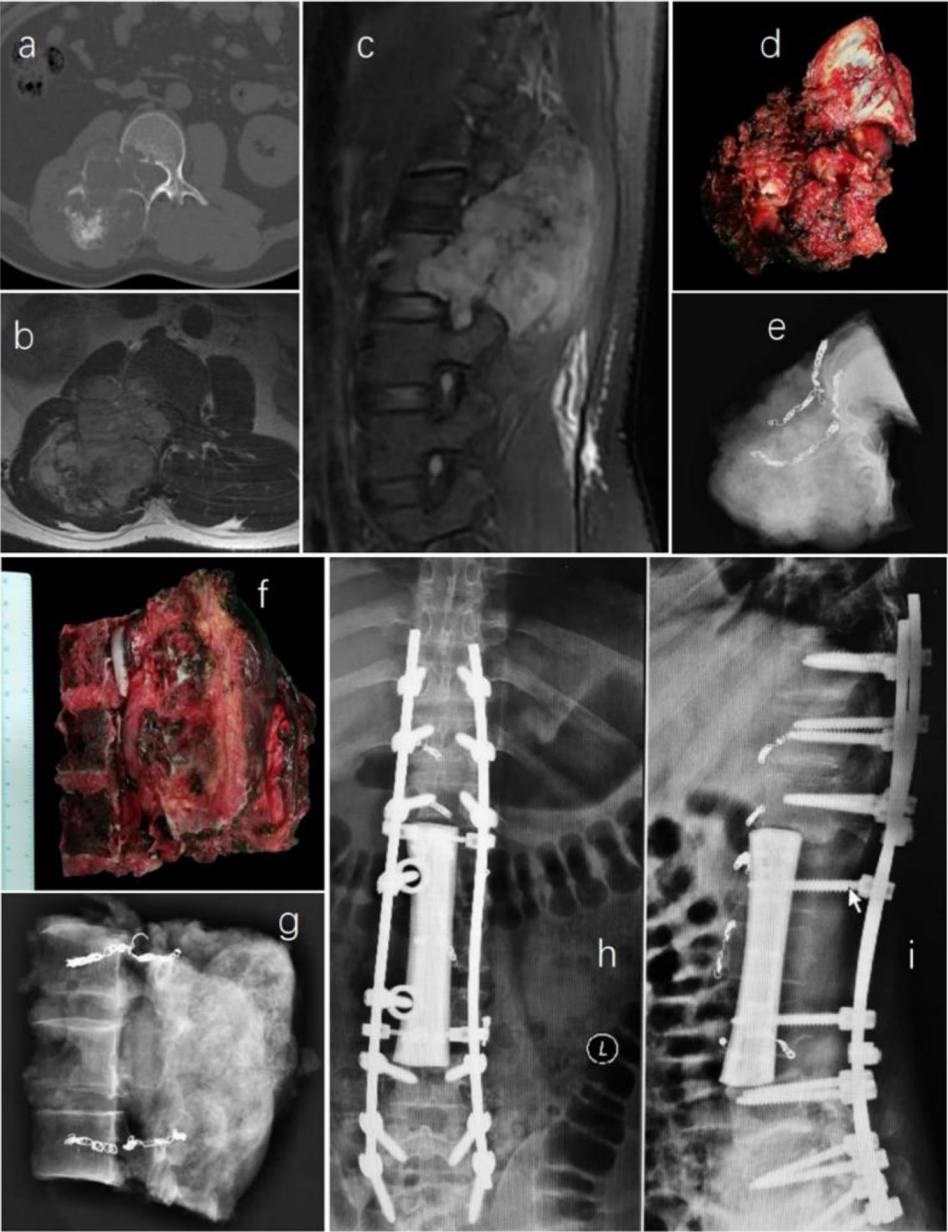
A 34-year-old male patient diagnosed with L1-3 chondrosarcoma (WBB stage: L1-3, sector 9–12, layer A-D) underwent en bloc resection with preservation of part of the vertebral body. The procedure involved a right retroperitoneal approach for initial tumor dissociation, followed by a posterior approach for sagittal resection of the involved vertebrae. Subsequently, a customized 3D-printed artificial vertebral body was implanted between T12 and L4. Preoperative images of the tumor are depicted in pictures **a**–**c**; pictures **d**–**g** show the specimen and its corresponding image; pictures h and i display postoperative images

**Fig. 2:**
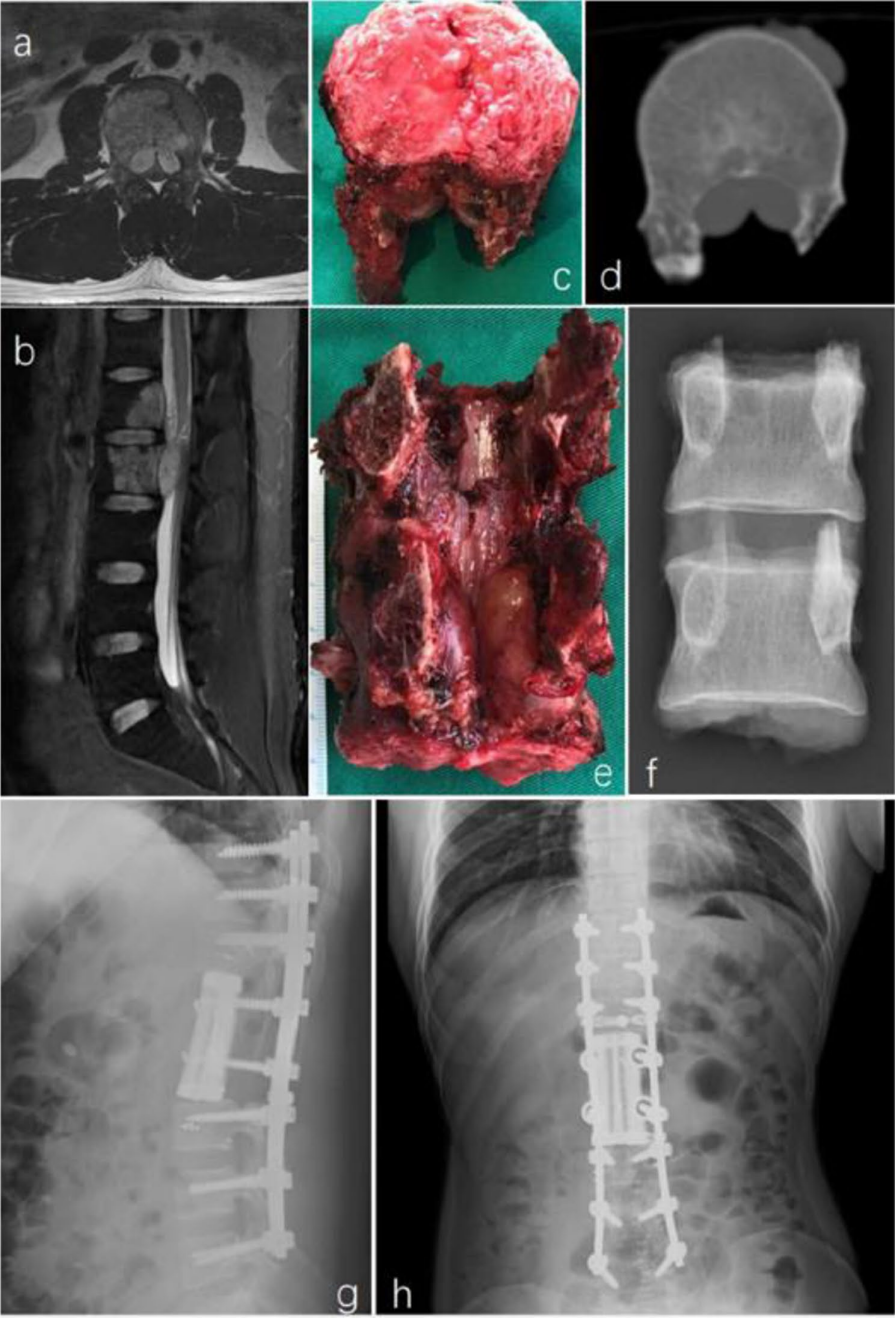
A 27-year-old male patient with L1-2 chordoma (WBB stage: L1-2 sector 3–10, layer B-D) underwent en bloc resection. A posterior approach was used.After resection of the posterior structures of L1 and L2 and the intervertebral disc between T12-L1 and L2-3, the L1-2 vertebral body and the tumor were resected en bloc. A 3D-printed artificial vertebral body was implanted between T12 and L3. Picture **a** and **b** were preoperative image of the tumor; picture **c**–**f** were the specimen and image of the it; picture **g** and **h** were postoperative image

Pre- and post-operative images, surgical specimens, and specimen images of en-bloc resection are depicted in Figs. 1 and 2.

### Data collection

A comprehensive review of the dataset encompassed demographic information, imaging findings, clinical characteristics, and surgical details. To assess spinal cord and dura compression, the Epidural Spinal Cord Compression (ESCC) score was employed, a metric previously validated for its reliability in evaluating intraspinal structure compression [24]. Complications were categorized as major or minor based on their impact on the patient’s recovery, following the classification by McDonnell et al. [25].

Patients underwent postoperative reexamination at our hospital or other medical facilities, with follow-up assessments conducted at three, six, and 12-months post-surgery, followed by annual evaluations. An important issue during follow-ups was the documentation of local tumor control. Tumor recurrence was confirmed through imaging studies and/or pathological assessments.

### Statistical analysis

Categorical variables were presented as numbers (percentages), while continuous variables were expressed as means (standard deviations, SD) or median values (interquartile range, IQR), depending on their adherence to normal distribution. Fisher’s exact test was applied for categorical variables, and for continuous variables, two-tailed unpaired Student’s t-test or Mann-Whitney U-test was employed, based on the normality of the distribution.

To analyze the occurrence of local recurrence over time, Kaplan-Meier survival curves were constructed, and log-rank tests were conducted. Data analysis was performed using SPSS 23 software (IBM, USA), with the significance level set at *P* < 0.05.

## Result

### Demographic and clinical characteristics

The final study cohort comprised 17 patients, consisting of 12 cases of chondrosarcoma and five cases of chordoma (Table 1). Among them, 8 were male (47.1%) and 9 were female (52.9%), with a mean age of 39.0 years (sd = 13.5) and a median hospital stay of 22 days (IQR = 18).

Of the cases, nine (52.9%) had tumors located in the thoracic spine, seven (41.2%) in the lumbar spine, and one case involved from T10 to L2. Notably, four cases exhibited recurrent lesions. All patients reported pain before surgery, with nine experiencing neurological dysfunction. Preoperative imaging indicated dural compression (ESCC scoring ≥ 1) in 13 cases (76.5%), spinal cord compression (ESCC scoring ≥ 2) in 9 cases (52.9%), and pedicle of vertebral arch invasion in 16 patients (94.1%).

Eleven cases were diagnosed with conventional chondrosarcoma, including 5 cases of WHO grade 1, 5 cases of WHO grade 2, and 1 case of WHO grade 3. The other case was confirmed clear cell chondrosarcoma.

### Surgical data

Single level resection was conducted in 6 cases (35.3%), while multiple level resection was undertaken in 11 cases (64.7%), resulting in an average of 2.7 segments resected (Table 1). In 5 cases (29.4%), intralesional piecemeal resection was employed. The remaining 12 patients (70.6%) underwent planned en bloc resection. The single posterior approach was utilized in 5 cases (29.4%), with 2 cases undergoing en bloc resection and 3 undergoing piecemeal resection. A combined approach was adopted in 12 patients (70.6%), of which 10 cases underwent en bloc resection, and 2 underwent piecemeal resection. The average surgical time was 684 min (sd = 287). Preoperative tumor supplying artery embolization was performed in 11 patients (64.7%), and the mean estimated blood loss was 2300 ml (sd = 1599).

**Table 1:** Demographic, clinical and surgical characteristics

Items	Values
Pathology (chondrosarcoma: chordoma)	12:5
Gender (male: female)	8:9
Age (years), Mean (SD)	39.0 (13.5)
Hospital stay (days), Median (IQR)	22 (18)
**Preoperative ESCC scoring, n (%)**	
0	4 (23.5)
1	4 (23.5)
2	4 (23.5)
3	5 (29.4)
**Preoperative neurological function, n (%)**	
Frankel A	0
Frankel B	1 (5.9)
Frankel C	0
Frankel D	8 (47.1)
Frankel E	8 (47.1)
**Location of tumor, n (%)**	
Thoracic spine	9 (52.9)
Lumbar spine	7 (41.2)
Thoracolumbar spine	1 (5.9)
**Surgical method, n (%)**	
En bloc resection	12 (70.6)
Piecemeal resection	5 (29.4)
**Surgical approach, n (%)**	
Posterior alone	5 (29.4)
Combined approach	12 (70.6)
Tumor-feeding vessels embolization, n (%)	11 (64.7)
Surgical duration (mins), Mean (SD)	684 (287)
Estimated blood loss (mL), Mean (SD)	2300 (1599)

ESCC Epidural spinal cord compression

### Perioperative complications

A total of 35 complications were documented (Table 2), averaging 2.06 perioperative complications per patient. The complications comprised 21 major and 14 minor occurrences. Fourteen patients (82.4%) experienced at least one perioperative complication, with major complications observed in 11 patients (64.7%). Specifically, the incidence of perioperative complications in the 12 chondrosarcoma patients was 83.3% (10/12), with a major complications rate of 66.7% (8/12). Among the 5 chordoma patients, the incidence of perioperative complications was 80% (4/5), with a major complications rate of 60% (3/5).

### Intraoperative complications

Major vascular injuries were observed in three patients (17.6%), involving the iliac vein, azygos vein, and segmental artery with the aorta. Immediate vascular suturing was performed in all cases. The average blood loss for these three patients was 4483 ml, significantly higher than the overall average (*P* = 0.05). The mean surgery duration for these cases was 870 min, marginally longer than the average (*P* = 0.222).

Dural tear with cerebrospinal fluid (CSF) leakage occurred in five cases (29.4%), and pleural injury occurred in two cases (11.8%). Most patients underwent suture repair for these complications.

### Early postoperative complications

Neurological deterioration was observed in five patients (29.4%). One patient developed progressive lower extremity muscle paralysis and hypoesthesia post-surgery. Although instant imaging examination did not reveal obvious spinal cord compression, symptoms worsened, ultimately leading to paraplegia. Four patients (23.5%) experienced decreased muscle strength or hypoesthesia, three of whom showed improvement with conservative treatment before discharge.

Pleural effusion requiring puncture drainage, closed thoracic drainage, or long-term indwelling thoracic drainage (> 7 days) occurred in five patients (29.4%), all of whom improved after puncture or drainage. Respiratory infection was noted in four cases (23.5%), with all patients recovering after anti-infection and oxygen inhalation.

Debridement was required in two patients (11.8%) due to poor wound healing, one of whom also had complications with CSF leakage. Deep venous thrombosis in the leg was identified in two patients (11.8%), with one patient succumbing to pulmonary thromboembolism three months after discharge. Three patients (17.6%) received multiple blood transfusions for anemia (≥ 2 times).

**Fig. 3:**
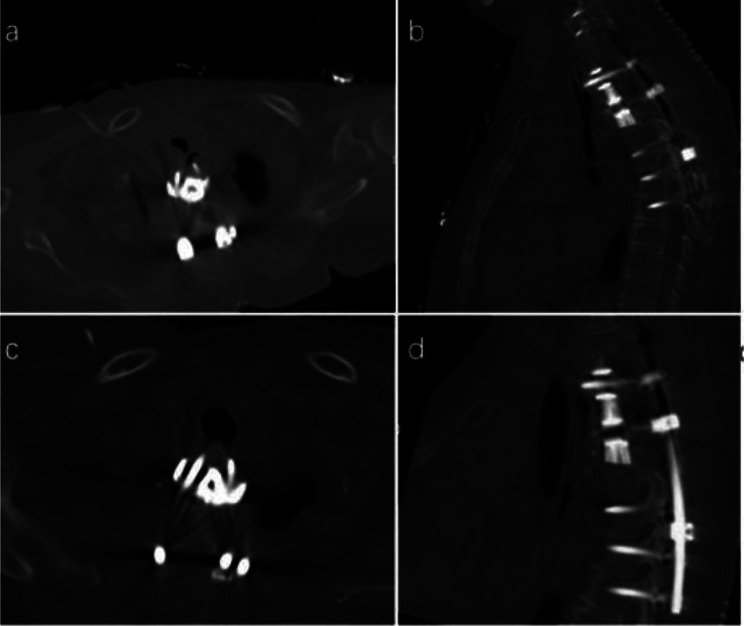
A 71-year-old female patient with T1-3 chordoma undergoing en bloc resection. (**a**, **b**) Illustrate the slightly longer screw at the upper left of the prosthesis, posing a potential risk of esophageal compression. (**c**, **d**) Display the adjusted screw following surgical intervention

**Fig. 4:**
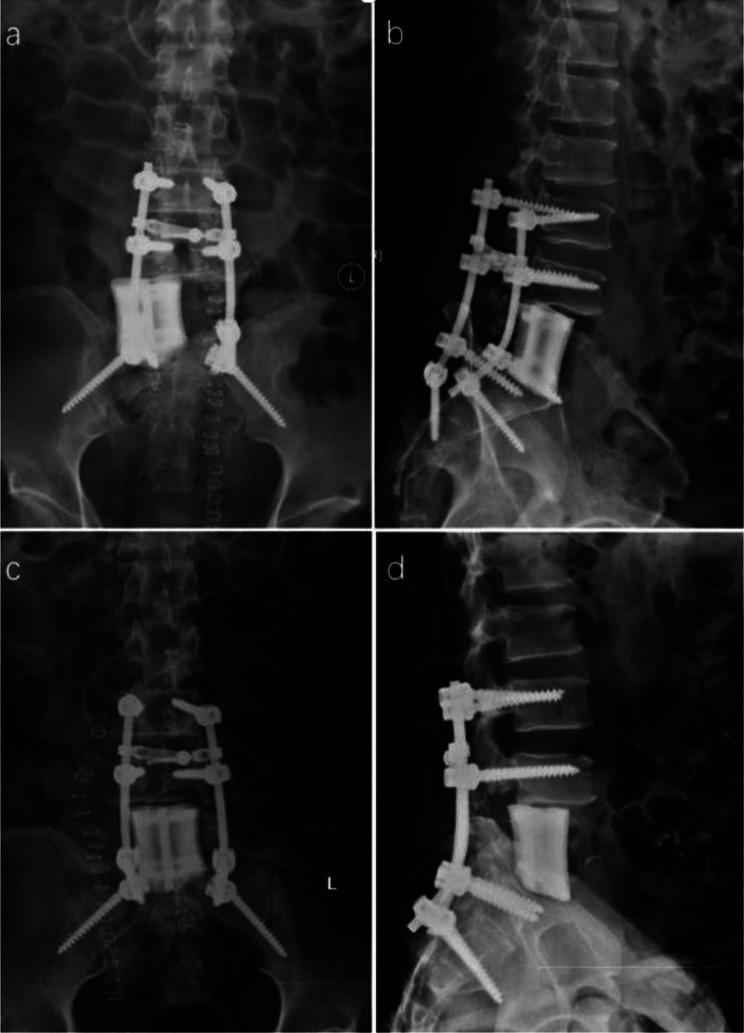
A 47-year-old male patient with L5 chondrosarcoma underwent en bloc resection. Picture **a** and **b** showed the prosthesis was displaced to the right side; picture **c** and **d** showed the adjusted prosthesis

Improper internal fixation was detected in two patients (11.8%) by postoperative imaging, leading to surgical adjustments within one week. In one case, postoperative CT revealed a slightly longer pedicle screw, suspected of compressing the esophagus, prompting replacement with a shorter pedicle screw (Fig. 3). In the other case, postoperative imaging showed internal fixation displacement, necessitating repositioning (Fig. 4).

Intracranial hemorrhage occurred in one patient, improving with conservative treatment. Additionally, one patient experienced postoperative chylous leakage, which resolved after one week of indwelling thoracic drainage, diet control, and parenteral nutrition support.

**Table 2:** Perioperative complications

Perioperative complications		n (%)	Treatment	Outcome
Major vascular injury		3 (17.6)	All vascular suture	All successful hemostasis
Dural tear with CSF leakage		5 (29.4)	4 dural sutures;1 drainage	All recovered
Pleural injury		2 (11.8)	All suture repairs	
Neurological deterioration	Paraplegia	1 (5.9)	Emergency surgical exploration	Paraplegia
	Decreased muscle strength or hypesthesia	4 (23.5)	Conservative treatment	3 improved; 1 unchanged before discharge
Pleural effusion		5 (29.4)	Puncture drainage or closed thoracic drainage	All recovered
Respiratory infection		4 (23.5)	Anti-infection and oxygen inhalation	All recovered
Wound healing problems		2 (11.8)	Debridement	All recovered
Deep venous thrombosis		2 (11.8)	Immobilization and anticoagulation	1 improved; 1 died due to PTE 3 months after discharge
Anemia		3 (17.6)	Blood transfusions	All improved
Improper internal fixation		2 (11.8)	Surgical adjustment	All recovered
Stroke		1 (5.9)	Conservative treatment	Improved
Chylous leakage		1 (5.9)	Long-term indwelling drainage, nutritional support, diet control and other treatments	Recovered

CSF stands for cerebrospinal fluid; PTE, pulmonary thromboembolism

### Follow-up

One patient succumbed to pulmonary embolism three months after discharge, while another patient was lost to follow-up after recurrence at 5 months post-surgery. The remaining patients were followed up for a minimum of 13 months, with a mean follow-up time of 55.3 months (sd = 22.7).

### Late complications

**Fig. 5:**
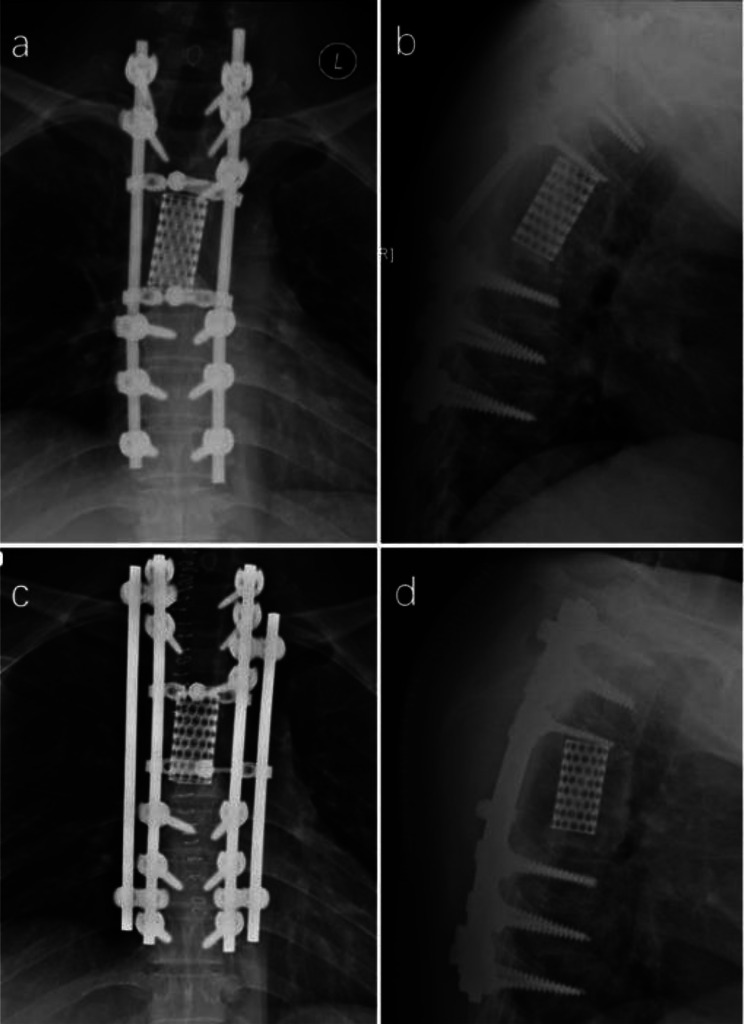
Fracture of the titanium rod observed 38 months after the operation in a 41-year-old male patient with thoracic chondrosarcoma. (**a**, **b**) Depict the fractured titanium rod; (**c**, **d**) Illustrate the internal fixation after revision surgery

One patient experienced postoperative internal fixation failure 38 months after surgery, specifically, a titanium alloy fixation rod fracture, necessitating revision surgery (Fig. 5).

### Local tumor control

**Fig. 6:**
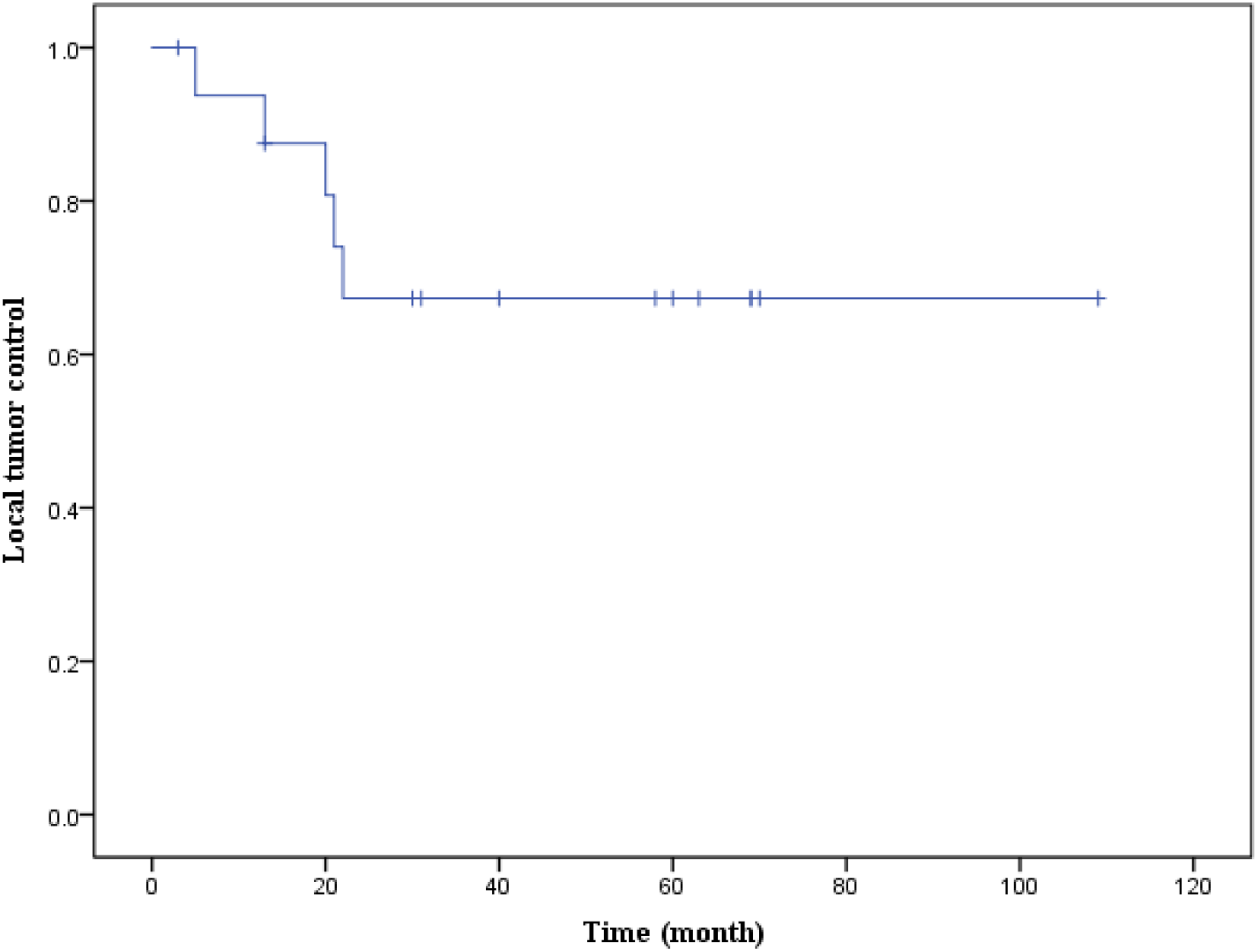
Kaplan-Meier Curve of local tumor control for all patients

Five patients experienced local recurrence during follow-ups (Table 3), with four cases involving chondrosarcoma patients, resulting in a local recurrence rate of 33.3% (4/12). The remaining case was a chordoma patient, yielding a local recurrence rate of 20% (1/5). The mean time to recurrence was 16.2 months (sd = 7.2), and the median recurrence time was 20 months (IQR = 12.5). The Kaplan-Meier curve of local tumor control is presented in Fig. 6.

**Fig. 7:**
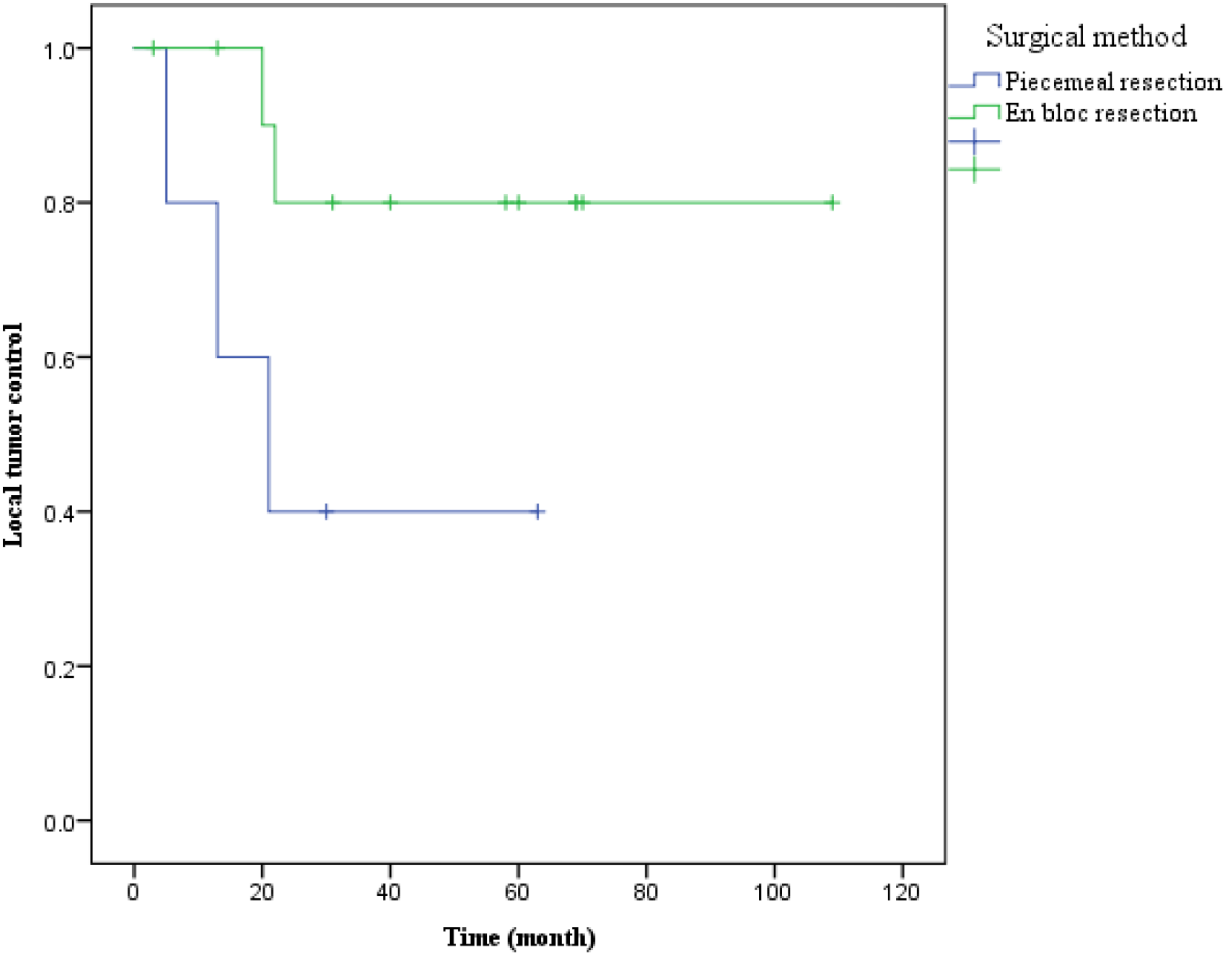
Kaplan-Meier curves comparing the local tumor control for patients who underwent en bloc resection versus piecemeal resection, indicating a potential advantage for en bloc resection, although statistical significance was not achieved (*P* = 0.067)

Among the local recurrent patients, two underwent en bloc resection, while three underwent piecemeal resection. Remarkably, the two cases of en bloc resection achieved a tumor-free margin. Patients treated with these two surgical methods did not exhibit significant differences in age, gender, resected segments, hospital stays, operation time, blood loss, and complication rates (Table 4). Although the local recurrence rate of en bloc resection was lower than piecemeal resection, the difference was not statistically significant (Fig. 7, log-rank test, *p* = 0.067).

**Table 3:** Characteristics of local recurrent cases

Case	Age/Gender	Classification of pathology	WBB stage	Surgical method	Recurrence time (m)	Duration of follow-up	Treatment	Outcome
1	47 M	Chondrosarcoma	L5 5–11 A-C	Piecemeal resection	21	46	Cytoreductive surgery and radiotherapy	Alive with neoplasm
2	38 M	Chondrosarcoma	L4 4–6 A-B	En bloc resection	20	66	Cytoreductive surgery	Alive with neoplasm
3	40 F	Chondrosarcoma	T9 3–10 A-D	En bloc resection	22	62	Radiotherapy	Alive with neoplasm
4	40 M	Chordoma	T12-L2 2–11 A-D	Piecemeal resection	13	44	Palliative surgery, radiotherapy and chemotherapy	Died of brain metastases 44 months after surgery
5	50 F	Chondrosarcoma	T11 2–11 A-D	Piecemeal resection	5	5	None	Loss of follow up after 5 months

**Table 4:** Comparison of two surgical methods

	En bloc resection	Piecemeal resection	P value
Gender M: F	5:7	3:2	0.62
Age, years (sd)	38.0 (15,6)	41.4 (9.4)	0.66
Median hospital stay, days (IQR)	23 (14.0)	19 (67.5)	0.916
Mean removed vertebral levels, n (sd)	2.0 (0.7)	2.4 (1.9)	0.68
Mean duration of surgery, min (sd)	717 (246)	617 (379)	0.54
Mean estimated blood loss, ml (sd)	2342 (1828)	2200 (1010)	0.84
Complication rate (%)	83.3	80.0	1.00
Major complication rate (%)	66.7	60.0	1.00

Among the three local recurrent patients who underwent cytoreductive surgery, radiotherapy (CyberKnife stereotactic radiotherapy), and/or chemotherapy, all were still alive with tumors at the time of the last follow-up.

For the other two patients, the tumors were well-controlled locally with no evidence of progression, but distant metastases developed after surgery. A 34-year-old man with lumbar chondrosarcoma was diagnosed with bilateral lung and pleural metastases six months after en bloc resection. Despite receiving chemotherapy and radiotherapy, the patient eventually succumbed to brain metastases 31 months after surgery. In another case, a 35-year-old female patient with L2-3 chondrosarcoma presented with an intraspinal lesion at the L5-S1 level 53 months after en bloc resection. Following the second surgery and radiotherapy, the patient recovered well, and there has been no evidence of tumor recurrence thus far.

## Discussion

En bloc resection remains the preferred treatment for patients diagnosed with chondrosarcoma and chordoma. However, achieving this procedure in spinal patients can be challenging due to the unique anatomical structure. Boriani and colleagues have proposed various procedures and techniques for achieving en-bloc resection in different situations [22, 23]. Nevertheless, there are exceptions where achieving this goal is technically impossible. Examples include tumors invading all bony structures surrounding the spinal cord or when a massive tumor involves vital yet unresectable organs, such as major vessels, trachea, or the spinal cord. In such cases, ensuring surgical safety becomes paramount, and performing piecemeal total resection becomes a necessary alternative.

In this study, the 12 cases of en bloc resection and 5 cases of intralesional resection demonstrated favorable safety outcomes, although differences in local tumor control were noted.

### Operative safety

Perioperative complications occurred in 82% of the patients, consistent with literature reports [13–18]. The surgical resection of thoracic and lumbar chondrosarcoma and chordoma is difficult and risky. The complications caused by en bloc resection or intralesional resection are different and the former has an increased rate [19]. Most of the complications have a good prognosis after suitable treatment, but a few complications can bring serious consequences.

In this study, three patients (17.6%) experienced large vessel injury, resulting in massive blood loss. The limited surgical field of view, particularly in lumbar cases undergoing a single posterior approach, increases the likelihood of injury to the prevertebral vessels. To mitigate this risk, the combined anterior and posterior approach can provide more space for tumor separation. Kawahara et al. have reported the safe completion of en bloc resection of L4 or L5 spinal tumors using the combined anterior and posterior approach [26]. When the tumor invades the vertebral body, especially extending beyond the anterior wall of the vertebral body (layer A according to the WBB surgical system), special attention is required to avoid vascular injury. To minimize the risk of severe blood loss, preoperative imaging evaluation, detailed surgical planning, and meticulous operative techniques are essential. Immediate suturing is typically employed for most vascular injuries, and if the complexity of the injury surpasses the capabilities of the same approach, an additional approach may be necessary.

Preoperative vascular embolization is an effective strategy to reduce intraoperative blood loss and operation time while improving tumor resectability [27]. Previous studies have demonstrated the high success rate and safety of preoperative embolization for thoracic and lumbar tumors [28].

Lower limb transient neurologic impairment is a common postoperative complication, with Shimizu et al. reporting up to 80% of patients experiencing lower extremity neurologic impairment after lumbar spine tumor en bloc resection [13]. The management of nerve roots varies, with direct ligation and transection often required for thoracic tumors, while lumbar tumors necessitate separation and preservation. As a result, traction of the nerve root during lumbar spine surgery can lead to decreased postoperative lower limb strength and paresthesia. Most patients can recover with conservative treatment over time.

However, in this study, a 47-year-old female chondrosarcoma patient developed paraplegia after surgery. The lesion was located in T4-5, and the preoperative Frankel classification was E grade. Despite undergoing staging combined anterior and posterior approach surgery with a total intraoperative bleeding volume of 650 ml, her lower limb muscle strength gradually decreased 15 h after the operation. Magnetic resonance imaging suggested the possibility of a perispinal hematoma, but emergency surgery did not reveal obvious spinal cord compression. Unfortunately, the patient ended up with paraplegia, which showed no improvement at the 69-month follow-up. However, no tumor recurrence was observed during the same period. The cause was considered to be delayed spinal cord ischemia, although no further evidence was available.

Pleural effusion and CSF leakage are also frequently encountered perioperative complications. Prophylactic closed thoracic drainage is deemed necessary for patients undergoing transthoracic procedures, those with pleural injury, or those with planned pleurotomy. Special consideration should be given to patients with a history of preoperative radiotherapy, recurrent tumors, and intraspinal lesions, as CSF leakage caused by dural adhesion may occur in these cases [29].

### Local tumor control

Spinal chondrosarcoma and chordoma exhibit limited sensitivity to conventional radiotherapy such as photon radiation and chemotherapy [30, 31]. Emerging research suggests that stereotactic photon beam therapy and proton beam therapy hold promise as alternative treatments [32–34]. Additionally, carbon ion therapy has shown potential, particularly for unresectable or residual sarcomas following incomplete surgery [35].

While these radiation therapies show promise, it is crucial to emphasize that en bloc resection remains the primary treatment choice, allowing for complete tumor removal. The most notable advantage of en bloc resection lies in avoiding tumor capsule penetration and tissue leakage, minimizing the risk of recurrence. In contrast, piecemeal resection is associated with tumor contamination and a higher recurrence risk [8]. Previous studies consistently demonstrate that en bloc resection reduces the local tumor recurrence rate of spinal chondrosarcoma and chordoma compared to intralesional resection [4, 6–8, 10–12, 36, 37].

In this study, 12 patients underwent en bloc resection, and 5 patients underwent intralesional total resection. The local recurrence rate aligned with previous reports: 16.7% (2/12) cases had local recurrence after en bloc resection, while 60% (3/5) experienced recurrence after piecemeal resection. Although the Kaplan-Meier curve did not show a significant difference in local control between the two groups, the advantage of en bloc resection in reducing local recurrence remains evident.

## Conclusion

In this research, the complication rates of the two surgical procedures were found to be similar. Considering both safety and local tumor control, we recommend en bloc resection as the primary choice for patients with chondrosarcoma/chordoma in the thoracic and lumbar regions who are eligible for this treatment.

## Data Availability

The datasets used and/or analyzed during the current study available from the corresponding author on reasonable request.

## Abbreviations

WBB: Weinstein-Borinani-Biagini
ESCC: Epidural spinal cord compression
CSF: cerebrospinal fluid
PTE: pulmonary thromboembolism
